# Retracted: Protective Effects of Pretreatment with Oleanolic Acid in Rats in the Acute Phase of Hepatic Ischemia-Reperfusion Injury: Role of the PI3K/Akt Pathway

**DOI:** 10.1155/2022/9825785

**Published:** 2022-04-26

**Authors:** Mediators of Inflammation

*Mediators of Inflammation* has retracted the article titled “Protective Effects of Pretreatment with Oleanolic Acid in Rats in the Acute Phase of Hepatic Ischemia-Reperfusion Injury: Role of the PI3K/Akt Pathway” [1] due to errors identified with the figures. Following the publication of the article, concerns have been identified as originally raised on Pubpeer [2]:

The concerns are related to Figures 4, 5 and 6:

- In Figure 4(a), 6 h p-PI3K displays a high amount of similarity with 3 h p-PI3K after narrowing. Additionally, the 0 h p-PI3K bands are also highly similar to the Prep p-PI3K bands after narrowing
- In Figure 5(a), the Prep AKT panel and the Prep GSK-3*β* panel in Figure 6(a) appear similar
- In Figure 6(a), the 0 hr p-GSK-3𝛽 and Prep p-GSK-3𝛽 of 6A are highly similar

The authors do not agree to the retraction, which has been agreed with the editorial board.

This notice replaces the corrigendum for the same issue [3], which was published in error.
